# Sensitivity to food additives, vaso-active amines and salicylates: a review of the evidence

**DOI:** 10.1186/s13601-015-0078-3

**Published:** 2015-10-13

**Authors:** Isabel J. Skypala, M. Williams, L. Reeves, R. Meyer, C. Venter

**Affiliations:** Royal Brompton and Harefield NHS Foundation Trust, Sydney Street, London, SW3 6NP UK; Somerset Partnership NHS Foundation Trust, Somerset, UK; Oxford Health NHS Foundation Trust, Oxford, UK; Great Ormond Street NHS Foundation Trust, London, UK; The David Hide Asthma and Allergy Research Centre, Isle of Wight, UK

**Keywords:** Food, Additives, Allergy, Histamine, Salicylate

## Abstract

Although there is considerable literature pertaining to IgE and non IgE-mediated food allergy, there is a paucity of information on non-immune mediated reactions to foods, other than metabolic disorders such as lactose intolerance. Food additives and naturally occurring ‘food chemicals’ have long been reported as having the potential to provoke symptoms in those who are more sensitive to their effects. Diets low in ‘food chemicals’ gained prominence in the 1970s and 1980s, and their popularity remains, although the evidence of their efficacy is very limited. This review focuses on the available evidence for the role and likely adverse effects of both added and natural ‘food chemicals’ including benzoate, sulphite, monosodium glutamate, vaso-active or biogenic amines and salicylate. Studies assessing the efficacy of the restriction of these substances in the diet have mainly been undertaken in adults, but the paper will also touch on the use of such diets in children. The difficulty of reviewing the available evidence is that few of the studies have been controlled and, for many, considerable time has elapsed since their publication. Meanwhile dietary patterns and habits have changed hugely in the interim, so the conclusions may not be relevant for our current dietary norms. The conclusion of the review is that there may be some benefit in the removal of an additive or a group of foods high in natural food chemicals from the diet for a limited period for certain individuals, providing the diagnostic pathway is followed and the foods are reintroduced back into the diet to assess for the efficacy of removal. However diets involving the removal of multiple additives and food chemicals have the very great potential to lead to nutritional deficiency especially in the paediatric population. Any dietary intervention, whether for the purposes of diagnosis or management of food allergy or food intolerance, should be adapted to the individual’s dietary habits and a suitably trained dietitian should ensure nutritional needs are met. Ultimately a healthy diet should be the aim for all patients presenting in the allergy clinic.

## Background

The recent food allergy guidelines from the European Academy of Allergy and Clinical Immunology give clear advice on how to diagnose an immune-mediated food allergy [1]. However, non-immune mediated reactions to foods are not addressed. Food additives and naturally occurring ‘food chemicals’ are attributed by many patients as the cause of their symptoms. Studies have assessed the potential for food additives and food chemicals to cause adverse symptoms, but robust research is difficult to conduct due to a number of factors: performing double blind placebo controlled challenges using foods or varying dosages of individual additives, setting inclusion criteria, standardizing the outcome and ruling out potential co- factors. Also the mechanisms involved have been poorly studied; although some preliminary data indicates the possible role of IgE involvement, many other mechanisms have also been proposed [2].

A vast range of adverse reactions have been attributed to the consumption of these added or natural ‘food chemicals’. The most frequent clinical features are chronic urticaria or angioedema [3], but symptoms can also include atopic eczema, [4] flushing, hypotension, abdominal pain, diarrhoea and asthmatic reactions, and occasionally severe or anaphylactoid or severe (anaphylactic) reactions [5, 6]. Due to the difficulty in diagnosing these conditions, the true prevalence of adverse reactions caused by food chemicals is unknown. A recent cross-sectional study from Japan estimated that the prevalence of chemical intolerances was 7.5 %, with fatigue, depressed mood and somatic symptoms positively correlated with a diagnosis of the condition [7]. Some groups appear to be at higher risk for example steroid-dependent asthmatics, those with marked airway hyper-responsiveness, and those with chronic asthma appear to be at greater risk of sulphite sensitivity [6]. Such prevalence data is even harder to come by in the paediatric population, where adverse reactions to food chemicals are poorly studied.

The range of the additives and natural substances potentially involved in non-immune mediated reactions in both children and adults makes it very difficult to undertake a systematic review of the literature. The following review provides an overview of the prevalence and foods thought to be involved in sensitivity to food additives, vaso-active amines and salicylates adults. It also considers separately the evidence for the dietary exclusion of these substances in the paediatric population.

## Food additives

### Sulphites

Sulphur dioxide has long been regarded as an air pollutant, but sensitivity to sulphites in foods was first described in 1973 (Kochen). Sulphites, readily metabolised to sulphate (SO_4_) by sulphite oxidase [8] mainly occur in the human diet due to the addition of sodium/potassium sulphite/metabisulphite to foods to prevent enzymatic and non-enzymatic browning, control oxidation and prevent bacterial growth. Sulphites added to foods will be present either as a free form mix of sulphur dioxide, bisulphite and sulphite, or bound to carbohydrates or proteins [9]. A plethora of reports in the 1980s demonstrated that sulphites in foods were provoking adverse reactions; by 1984 the US Food and Drug Administration had received more than 250 reports of suspected sulphite reactions including six deaths [10]. Thus both the European Union and the US Food and Drug Administration have regulated the use of sulphites, requiring foods containing more than 10 ppm (10 mg/kg) to be labelled, but there is significant variation between levels for the same foods [11–15]. Foods containing a high level of free form sulphite are more likely to provoke a reaction and this dictates how much sulphite can be added [16]. Foods usually containing significant levels of added sulphite include cider, white wine, and dried fruits—see Table 1 [5, 17, 118].

**Table 1:** Foods more likely to contain high levels of natural or added sulphites, benzoates and monosodium glutamate

	Sulphites (E220–E227) [11, 17, 118, 119]	Benzoates (E210–E219) [30, 31, 33, 119, 120]	Monosodium glutamate (E621–E623, E627, E635) [49, 121]
Meat, poultry and seafood	Prawns, lobster, dried salt cod, crab sticks, squid, meat burger, sausages	Dishes with a spicy sauce, ready to eat meals containing benzoates	Fish sauce
Milk and eggs		Yoghurt, cheese	Parmesan cheese
Fruits	Dried apricots, sultanas, figs, prunes, dates, dried banana, candied or glace fruit desiccated coconut, currants	Cranberries, bilberries, prunes, papaya, dried fruit, avocado	
Vegetables, nuts, seeds and savoury snacks	Dried mushrooms and other fungi, frozen, tinned or vacuum packed potatoes, French fries, instant mash, gnocchi, potato cakes, potato croquettes, vegetarian burgers and sausages, tinned asparagus, broad beans, French beans, chestnuts, walnuts	Pumpkin, kidney beans, soy beans, soy flour, broccoli, spinach, baked beans in tomato/spicy sauce, dry roasted and spicy nuts, Bombay mix, crisps (except ready salted), potato or corn snacks,	Mushrooms, spinach, savoury snacks, crisps
Condiments and miscellaneous	Horseradish sauce, caramel colouring (E150)	Curry powder, allspice, mixed spice, nutmeg, clove, cinnamon, chocolate, cocoa, ketchup, soy sauce, Worcestershire sauce, salad dressing, salad cream, mayonnaise, jam, pickles	Soups, stock, gravy, rubs, coatings, ready-meals, soy sauce, black bean sauce, oyster sauce, tomato sauce, miso, marmite, instant rice and noodle dishes
Drinks	Cider, wine, beer, fruit squash and cordials, soft drinks, grape juice, fruit juice drinks, cola drinks	Tea, squash, cordial, carbonated drinks, milkshake syrup, beer, ready-to-drink alcohol and mixers, spirits with added spices	

Foods with added sulphite, benzoates and MSG will vary from country to country

Sensitivity to sulphites mainly affects patients with asthma, especially those with severe steroid-dependant asthma. Bush and colleagues undertook single-blind challenges with encapsulated sodium metabisulphite in 203 asthmatic subjects and reported a prevalence of 3.9 % in asthmatic patients, with those who were steroid dependant being most at risk [18]. Buckley et al., using similar methodology, found a similar prevalence of 4.6 % in a large cohort of asthmatic patients [19]. A recent review by Vally and Misso suggested that 3–10 % of asthmatics experience symptoms on exposure to ingested sulphites [5]. An analysis of sulphite-sensitive cases in Korea found that two phenotypes of sulphite sensitivity existed, those with sulphite sensitive asthma was the most common, affecting two-thirds of their cohort, with the remainder having sulphite-sensitive urticaria [20]. Sulphites can cause urticaria and angio-oedema both through topical application of sulphite containing cosmetics or skin reactions following oral ingestion of sulphite [5]. Other reactions are less common but include anaphylaxis [6] and rhinitis [21].

It is postulated that the inhalation of sulphur dioxide, generated from ingested sulphites may cause respiratory symptoms [6, 22]. Other mechanisms suggested include IgE-mediated reactions [6, 23, 24], a deficiency of sulphite oxidase [25], and a role for leukotrienes [26]. Due to the unpredictability of the level of available sulphite in different foods, and because sulphite sensitive individuals will not all respond in the same way to sulphite-containing foods, few trials have used foods when evaluating sulphite sensitivity [27]. Taylor and colleagues undertook double-blind, placebo-controlled food challenges (DBPCFC) with foods known to be high in sulphites (lettuce, dehydrated potato, shrimp, dried apricots, white grape juice) in eight subjects whose sulphite sensitivity was established through the use of DBPCFC with encapsulated potassium metabisulphite [28]. Four patients failed to respond to any of the challenges, four responded to sulphited lettuce and there was a varying degree of response to the other foods. Using DBPCFC Vally and Thompson found only 16 % of wine sensitive asthmatics responded to sulphite additives in wine [29].

### Benzoates

Benzoic acid is produced by many plants and has also been detected in animals, so is present in many foods, including berries and milk products, usually in relatively low concentrations of up to 40 mg/kg [30–32]. Benzoate can also be a product of digestion, e.g. cinnamic acid from cinnamon is oxidised to a benzoate salt in the liver [33]. Benzoates are also added in much higher concentrations to soft drinks, jams, sweets, chocolates, ice creams, pickles, baked goods due to their antimicrobial properties—see Table 1 [34, 35].

Benzoates have been linked to chronic urticaria, asthma, atopic dermatitis, rhinitis and anaphylaxis although there is limited good quality evidence to support these findings. Wuthrich et al. proposed that benzoates may be involved via a type IV sensitisation causing allergic contact dermatitis [36] and Van Bever et al. used DBPCFC to show that benzoates may cause atopic dermatitis [37]. Worm et al. also undertook DBPCFC to show a late phase response in atopic dermatitis, but the capsules used contained a mixture of additives including benzoates rather than benzoates alone [38]. In a more recent study using a DBPCFC to a mixture of food additives including benzoates, Park et al. showed they had no effect on atopic dermatitis [39].

In another study, Weber et al. hypothesised that benzoates could exacerbate asthma, however in the DBPCFC study, only 1/43 subjects had a positive result, with individual testing negative on re-challenge 2 years later [40]. In 1981 Tarlo et al. found only 1/28 subjects with chronic asthma reacted to benzoates, but did not respond symptomatically to a benzoate-free diet [41]. Persistent rhinitis has also been linked to consumption of benzoates; Pacor et al. showed that 8.8 % (20 subjects) reacted to a DBPCFC challenge to monosodium benzoate. The subjects with a positive challenge had previously reported an improvement in symptoms within in 1 month of using an additive free diet and a return in rhinitis symptoms with the adoption of a food additive rich diet [42].

Only two robust studies have assessed the link between chronic urticaria and benzoates using blinded placebo controlled trials. Ortolani et al. reported that 3/72 (4 %) patients reacted to benzoates [43], but Simon et al. found no positive reactions in their study [44]. Several studies using dietary elimination in chronic urticaria showed positive outcomes to benzoates, but they had either no clear outcome measures, lack of blinding and/or no placebo [45–47]. Rajan and colleagues showed that although 2/100 patients had a positive response to single-blinded challenges with the individual additives including sodium benzoate, neither responded to DBPCFC [48]. They concluded that food and drug additives were a rare cause of chronic urticaria and that avoidance should not be recommended. It is unclear why these studies offer contradictory evidence and also whether natural benzoate is also likely to provoke adverse reactions in the same way that added benzoate might.

### Monosodium glutamate

Monosodium glutamate (MSG—E621) is a common added ingredient to savoury foods. Glutamate also occurs naturally in other foods, with ripening of fruits such as tomatoes and the curing of meat such as ham, being associated with an increase in the free amino acids such as glutamate [49]—see Table 1. The l-glutamate derived from dried kelp or seaweed, was originally used to make MSG prior to synthetic derivatives being developed [50]. Originally described as ‘Chinese Restaurant Syndrome’ in 1968 [51], results from studies utilising DBPCFC have suggested some individuals could experience symptoms from the ingestion of MSG, although only in quantities greater than the normal dietary intake [50, 52]. Other studies have found MSG does not provoke any symptoms [53]. Geha et al. conducted a DBPC crossover study on 130 subjects with reported MSG sensitivity; 2/130 responded to a large dose given without food, with no responses noted when MSG was given with food [54]. Although the glutamate in MSG is chemically indistinguishable from glutamate present in food proteins and is metabolized in the same way [55], it is currently unknown whether foods high in natural bound or free form glutamate can cause the same symptoms as MSG.

This additive has been linked to asthma, headache, urticaria and angioedema, rhinitis, psychiatric disorders and convulsions [56, 57]. A link with asthma was first noted by Allen and Baker [58] although subsequent studies have shown mixed outcomes. Schwartzstein et al. reported that DBPC with MSG were negative in 12 subjects with asthma [59]. Similarly, Woods et al. reported no changes in forced expired volume in one second (FEV1) values and no immediate or late asthmatic reactions in their cohort of 12 subjects [60]. Germano et al. found 1/30 participants experienced a significant reduction in FEV1 during single blind provocation, but this was not reproducible during DBPC challenges [61].

Persistent rhinitis linked to MSG has been demonstrated in DBPC challenges by Asero, but only in a handful of subjects [62]. Pacor et al. found 8/226 non-atopic subjects exhibiting subjective symptoms of rhinitis after a DBPC MSG challenge, and concluded that reactions to additives could precipitate rhinitis in some patients [42]. Case reports have linked MSG to urticaria [21], but Simon et al. showed only 2/65 subjects had a positive response in single blinded challenge, neither of whom were positive to a DBPC challenge [44].

Headache has been the most commonly reported symptom in relation to MSG [63] Yang et al. undertook DBPCFC in 61 subjects with self-reported sensitivity to MSG; 18/61 had no response, 21/61 had a placebo response and 22/61 a positive response to the active challenge only. On re-challenge a threshold dose of 2.5 g MSG was established [64]. Baad-Hansen et al. conducted a DBPCFC in 14 healthy individuals and reported a significant increase in reported headache and pericranial muscle tenderness after taking an individually tailored dose of MSG (150 mg/kg) [65]. However, despite these studies, an authoritative view is that the weight of the literature does not presently support the use of an MSG free diet for patients with chronic headaches [50].

## Naturally occurring ‘food chemicals’

### Biogenic/vasoactive amines

Biogenic or vasoactive amines are produced by bacteria during fermentation, storage or decay [6, 66–68]. They include beta-phenylethylamine, tyramine, tryptamine, putrescine, cadaverine, spermine and spermidine, but histamine is the one most frequently linked to food-related symptoms. When plasma histamine levels are raised above the normal range (0.3–1.0 ng/mL) this produces certain effects. For example a level of 1–2 ng/mL causes increased gastric acid secretion and heart rate, with, flushing, headache, urticaria, pruritus and tachycardia occurring at a level of 3–5 ng/mL), bronchospasm at a level of 7–12 ng/mL and cardiac arrest occurring at levels of 100 ng/mL [6]. Thus large amounts of ingested histamine can cause significant symptoms in otherwise well individuals. For example symptoms of flushing, sweating, urticaria, GI symptoms, palpitations and in severe cases bronchospasm may occur following the consumption of spoiled fish [69]. This condition, known as scombroid poisoning, occurs due to the high level of histidine in certain fish species being converted into histamine by marine bacteria [70]. Due to the nature of the symptoms caused, reactions involving vasoactive amines may therefore be incorrectly diagnosed as a food allergy.

Although 75 mg of liquid histamine can provoke symptoms in healthy volunteers [71], defining a safe threshold level for sensitive individuals is difficult [72]. Foods generally considered to contain high levels of vaso-active amines are shown in Table 2, however the amounts of histamine found in different foods varies according to the type of bacteria, food composition and conditions for fermentation [73]. Bodmer et al. found that mean levels of histamine were 3.63 mg/L for French wines, 2.19 mg/L for Italian wines and 5.02 mg/L for Spanish wines [68]. Symptoms to wine may not always be a reliable indicator of sensitivity to histamine. In a placebo-controlled study, Kanny et al. found no correlation between wine histamine content and wine intolerance, and concluded that other vaso-active amines or sulphites may be more relevant in intolerance to wine [74]. It has been proposed that other foods may be able to cause histamine release directly from tissue mast cells [6] although evidence for this is lacking [75].

**Table 2:** Foods more likely to contain high levels of vaso-active amines and salicylate

	Vaso-active amines [6, 75, 122–126]	Salicylate [85, 87–90, 97]
Meat, poultry and seafood	All cured meat especially pork products e.g. ham, salami, pepperoni, game, bacon, sausages, fresh pork, fresh or canned tuna, canned sardines, anchovies, mackerel, salmon, herring, processed fish products (fish pastes, smoked, dried or pickled fish), fish sauce	
Milk and eggs	Blue cheese, parmesan, brie, camembert, emmenthal, old gouda, cheddar cheese and other hard cheeses	
Fruits	Oranges, bananas, tangerines, pineapple, grapes, strawberries	Granny smith apples, cherries, strawberries, currants, raisins, kiwi, Gala melon, peaches and nectarines, raspberries
Vegetables, nuts, seeds and savoury snacks	Tomatoes, pickled cabbage, aubergine, spinach, broad beans, peanuts, tree nuts	Asparagus, sweet corn, raw tomatoes, tomato puree
Condiments and miscellaneous	Fermented soy products including miso and tempeh	Ginger, mixed herbs, mustard, oregano, curry powder, black pepper, cardamom pods, cinnamon, cumin, fenugreek, mint, nutmeg, paprika, rosemary, thyme, turmeric, liquorice, peppermint, Worcestershire sauce, honey, tomato ketchup
Drinks	Green tea, champagne, coffee, cocoa, chocolate, wine, beer, fresh fruit juices, smoothies	Coffee, pineapple juice, cider, Benedictine liqueur, lemon tea, black tea, apple juice, cranberry juice, orange juice, tomato juice, fizzy drinks, Drambui liqueur, wine, rum

Wantke et al. found a diet low in vasoactive amines alleviated chronic headache in 73 % of patients [76]. Another study reported that 27/44 (61 %) of subjects had a significant improvement in idiopathic urticaria, angio-oedema and pruritus on a diet low in dietary amines, although foods containing additives or high in natural salicylate were also restricted [72]. King and colleagues reported subjects with chronic urticaria/angioedema had a marginally significant reduction in their use of antihistamines on a histamine-reducing diet compared to a control group who eliminated artificial sweeteners from their diet. However response to the diet varied and the numbers in each group were very low [77]. Another study found that 58 % of adult patients with irritable bowel syndrome (IBS) considered foods rich in vasoactive amines, such as wine, beer, salami and cheese, to be a cause of their symptoms [78].

The diagnosis of sensitivity to vasoactive amines is usually made through history and dietary exclusion; however some studies have suggested that the measurement of diamine oxidase (DAO) levels may be helpful. One study found a DAO level <3 kU/mL was associated with reported symptoms to high histamine foods, whereas a level of >10 kU/mL indicated histamine intolerance was unlikely [6]. Patients with chronic idiopathic urticaria and GI symptoms have been shown to have reduced DAO activity [79, 80]. Another study reported that the size of the skin prick test wheal to histamine after 50 min, the ‘histamine 50-skin-prick test’, was a useful diagnostic indicator; 82 % of subjects with histamine intolerance maintained a wheal size greater than 3 mm compared with 18 % of controls [81].

### Salicylates

Salicylate intolerance has been defined as ‘a nonspecific antigen-induced pseudo-allergic hypersensitivity reaction to salicylic acid, its derivatives or other related organic or inorganic acids of similar chemical structure’ [82]. Salicylic acid is widely distributed in plant foods and, like its synthetic counterpart acetyl salicylate (Aspirin™) has anti-inflammatory activity. Acetyl salicylate is a potent inhibitor of COX-1, an isoform of the enzyme cyclooxygenase (COX), which prevents the conversion of arachidonic acid to cyclic prostanoids, whereas salicylic acid inhibits COX-2 gene expression [83, 84]. A diet rich in spices, which contain high levels of in salicylic acid, has been linked to lower levels of colorectal cancer in rural inhabitants of Southern India [85], although caution has been advised with regard to an over-emphasis on the importance of dietary salicylates compared with other bioactive plant phenolics in the diet [86].

The reported level of salicylates in different foods is highly variable between studies. Swain et al. published the salicylate content of 333 foods [87] and these data continue to underpin dietary salicylate avoidance advice. Subsequent studies have reported different results for the same foods [88–90], possibly due to diverse analytical methods and the origin, processing and storage of the foods tested [91–93]. Table 2 gives a list of high salicylate foods based on all of the published data. There is also uncertainty regarding the bioavailability of dietary salicylate; Jansen et al. reported that urinary salicylate excretion showed that the bioavailability of dietary salicylate in most people is low [94], whereas Lawrence and colleagues showed bioavailability to be high [95]. Curry spices, universally agreed as being high in natural salicylate, not only have a high bioavailability, they also inhibit both COX-1 and COX-2, which may indicate they could be a more important dietary factor than other high salicylate foods [85].

Baenkler has proposed that 2.5 % of Europeans may have salicylate sensitivity [96], but the evidence on which this assertion is based is sparse. Aspirin exacerbated respiratory disease (AERD), an inflammatory disease exacerbated by aspirin or other non-steroidal anti-inflammatory drugs (NSAIDS), usually presents in adult life, and affects up to 20 % of asthmatic subjects and 40 % of those with nasal polyps [97]. It has been shown that AERD subjects have down regulation of COX-2 expression in nasal polyps [98]^,^ so it could be hypothesised that such individuals might be more likely to be affected by dietary salicylate but there is conflicting evidence. Corder and Buckley reported that a challenge with salicylic acid had a significant effect on the lung function, whereas Dahlen et al. found no such reactions [99, 100].

Salicylate intolerance has also been proposed as a cause of other conditions; Raithel et al. proposed that 2–7 % of all patients with inflammatory bowel syndrome and food allergies could be affected by salicylate intolerance [82]. Gibson and Barrett suggest that since there are no published studies demonstrating efficacy of salicylate avoidance in gut disease, they recommend the dietary restriction of fermentable oligosaccharides, disaccharides, monosaccharides and polyols (FODMAPs™) as first line therapy for the majority of patients with functional bowel symptoms [101, 102].

## Chemical diets in children

Evidence for the efficacy of diets free from individual additives or high levels of natural food chemicals in children is sparse. There is a lack of convincing published data on the effect of the avoidance of food additives, although Supramaniam et al. demonstrated that 3/36 (10 %) children exhibited urticaria or angioedema in response to MSG [103], and avoidance of MSG was helpful in the management of 86 % of children aged 2–16 years with cyclical vomiting syndrome (a variant of migraine in children) [104]. A study on benzoates reported that 3/25 children reacted to sodium benzoate, but the reaction criteria were unclear and no reactions occurred during placebo challenges [37]. More rigorous studies concluded sodium benzoate or artificial colours (or both) in the diet may result in increased hyperactivity in young children [105], although genetic variants associated with the breakdown and clearance of histamine were found to moderate the impact of additives on behaviour [106].

Studies on sulphite intolerance in asthmatic children also appear to demonstrate some degree of effect. Sanz et al. [107] found that 4/20 children with asthma (7–14 years of age) had a positive reaction on double-blind challenge to sodium metabisulphite solution. However, in a subsequent study performed on 5 children with challenge confirmed sulphite intolerance; with a dose of 1.5 mg of oral cyanocobalamin followed by a repeated sodium metabisulphite challenge only resulted in bronchospasm in 1/5 of the children. [108]. There is also limited evidence for the efficacy of diets low in natural food chemicals. In 2013, Hoffmann et al. [109] reported that 14/394 children who presented with gastrointestinal discomfort, had both low levels of DAO and subsequently improved on a diet excluding foods high in histamine. However, the improvement was subjective, being based on a non-validated questionnaire and the dietary exclusion was not blinded. Another study has also shown that a low histamine or histamine-free diet improves symptoms in children with DAO levels <10 kU/mL, but only subjective symptoms were noted [110]. An extensive literature review yielded no prospective randomised controlled studies establishing the link between hypersensitivity reactions in children and salicylates.

Despite this lack of evidence, there has been a rise in the use of low chemical diets for children for a variety of conditions, in particular for atopic dermatitis, gastrointestinal symptoms and hyperactivity [111]. These diets typically remove foods high in salicylates, histamine, sulphites and MSG from the diet and may be used in conjunction with the dietary elimination of milk, soy, egg and wheat. A retrospective case review by Gray et al. found that almost half of a group of 74 children placed on a diet that eliminated foods naturally high in salicylates suffered adverse outcomes, including nutritional deficiencies, food aversion and eating disorders [112]. Growth velocity is highest within the first years of life and a failure to achieve this potential has a far reaching impact on development and later health [113, 114]. Many of the foods that contain both additives and naturally occurring food chemicals which are listed in Tables 1 and 2, contribute essential nutrients for growth and development. In addition, a lack of taste exposure during early infancy has been shown to lead to feeding difficulties in later life [115]. Therefore unwarranted dietary eliminations of these chemicals should be avoided in young children unless there is exceptional evidence as to their efficacy.

## Conclusion

The lack of well-designed placebo-controlled studies investigating the role of many of these substances makes it impossible to provide evidence-based advice for the efficacy of the removal of food additives, histamine and salicylates from the diet. No convincing data exists on the impact of food chemicals on atopic disorders in children, and in light of the importance of growth and development, these diets should be approached with caution in the paediatric population. For adults, the nutritional aspects might appear to be less of an issue, but significant dietary restrictions can affect nutritional status at any age [116]. Thus, the risk–benefit ratio should be considered when contemplating the dietary exclusion of multiple foods, particularly those foods which may confer some health benefit such as foods high in natural salicylates. The literature suggests that the removal of high histamine foods or those containing high levels of sulphites may provide some symptom relief. There is far less evidence on the beneficial effect of restricting other additives in the diet and no published evidence at all for the efficacy of low salicylate diets.

Studies which utilise DBPCFC have rarely shown food additives to be the cause of pseudoallergic symptoms. However, although DBPCFC with the individual additives or pure forms of histamine are ideal, they do not provide a ‘real-life’ scenario, where the additive or natural food chemical will be consumed as part of the food or food matrix. Ideally the potential cause and effect of foods should be reviewed on an individual basis and any diagnostic diet tailored to the individual, taking account of their symptoms and particular foods suspected, rather than adopting a blanket approach of a few foods diet. There are some foods which are a rich source of additives and/or natural food chemicals (Table 3) and so a limited removal of a few ‘high’ risk foods in adults might be a good first step to determine whether diet plays a role in symptoms.

**Table 3:** Foods likely to be high in added and/or natural ‘food chemicals’

Food	Amine	Glutamate	Salicylate	Sulphite	Benzoate
Cheese	✓	✓			✓
Wine	✓		✓	✓	
Soy	✓	✓			✓
Tea		✓	✓		
Tomatoes	✓	✓	✓		✓
Herbs and spices			✓		✓
Strawberries and pineapple	✓		✓		
Spinach	✓	✓			✓
Worcestershire sauce		✓	✓		✓
Dried fruit			✓	✓	✓

The elimination of an additive or food chemical often requires a major change to the whole diet, so any benefits arising from the intervention, may be due to lifestyle changes incurred as a result of an improvement in diet, rather than to the removal of the offending additive. In addition, the placebo effects of dietary interventions are as yet an unknown quantity, and require further study. The importance of healthy diets and diversity of foodstuffs has recently been demonstrated in infants and young children, but the lifelong effects may also be important in adults [117]. A healthy diet will usually contain lower levels of additives and therefore a pragmatic approach should focus on improving the nutritional quality of the diet alongside any specific dietary exclusion is an important goal in all dietary interventions for non-immune mediated reactions to foods.
